# High-energy storage capacity of cellulose nanofiber supercapacitors using bound water

**DOI:** 10.1038/s41598-023-43222-7

**Published:** 2023-10-03

**Authors:** Mikio Fukuhara, Tomonori Yokotsuka, Takuya Takashina, Nobuhisa Fujima, Masahiro Morita, Tatsunori Ito, Takeshi Nakatani, Toshiyuki Hashida

**Affiliations:** 1 New Industry Creation Hatchery Center, Tohoku University, Sendai, 980-8579 Japan; 2 Instrumental Analysis Group, Graduate School of Engineering, Tohoku University, Sendai, 980-8579 Japan; 3 Faculty of Engineering, Shizuoka University, Hamamatsu, 432-8561 Japan; 4 Fuji Innovative Materials Research Laboratory, Nippon Paper Industries, Co. Ltd, Fuji, 417-8520 Japan

**Keywords:** Biotechnology, Structural biology, Energy science and technology, Materials science

## Abstract

The performance of electric double-layer capacitors and lithium-ion batteries deteriorates with increasing humidity. The desirable effect of bound water on the energy-storage properties of physically dry cellulose nanofiber (Na-ACF) supercapacitors with sodium (Na) carboxylate radicals was investigated using infrared and near-infrared spectroscopy, and nuclear magnetic resonance spectroscopy, alternating current impedance analyses, and first-principles calculations. The storage capacity decreased gradually upon heating to 423 K and reached zero upon exceeding 483 K, accompanied by increasing electrical resistance, forming a distorted semicircle in Nyquist diagram and drawing the phase angle to zero in Bode diagram. This is attributed to the water in the hydration gel bound to the Na^+^-ions that cross-link the cellulose chains, evaporating as the temperature increases, and finally becoming Na_2_O. The increased band-gap energy from the increase in bound water prevents leakage from the supercapacitor. In contrast to ordinary batteries, bound water is necessary for developing Na-ACF supercapacitors.

## Introduction

The electronic properties of biodegradable cellulose nanofiber (CNF), characterised by thermal stability, high durability, and low-weight, have received significant attention with the advent of high- energy-storage supercapacitors^1,2^ and *n*-type semiconductors with N-type negative resistance^3^. We developed physical dry cellulose nanofiber (ACF) supercapacitors with high voltage-fast-charging performance of up to 500 V based on an electric double-layer model in a C_12_H_17_O_11_Na electrolyte, using both the quantum size effect and the offset effect of the positive charges on uneven solid electrolyte material surfaces^1,2^. The high-energy storage capacity of Na-ACF (1416.7 mJ/m^2^) which is similar to that of amorphous alumina supercapacitors (1710.3 mJ/m^2^)^4^, is attributed to the higher work functions of −22.5 eV, owing to the quantum-size effect from low convexity, an electrostatic effect from the appearance of localised electrons near the sodium (Na^+^) ions. ACF supercapacitors differ from conventional wet cells such as electric double-layer capacitors and lithium-ion batteries that are controlled by ion diffusion^5,6^. The performance of these batteries deteriorates with upon exposure to moisture and humidity^7,8^. However, in the present study, the desirable effect of water bound to Na on the energy storage properties was investigated based on the results of infrared (IR) and near-infrared (NIR) spectroscopy, nuclear magnetic resonance (NMR) spectroscopy, alternative current (AC) impedance analyses, and first-principles calculations. Understanding the formation and dissociation of hydrogels composed of complex compounds with bound water is crucial in botanical physical science. Although the hydration and dissociation processes of alkali metal hydroxides are key aspects of electronic, biological, and atmospheric sciences for living beings, they are not comprehensively understood^9,10^. In this study, we used ^1^H, ^13^C, and ^23^Na solid-state NMR spectroscopy to examine the structure and dynamic properties of Na^+^ ions and the water molecules surrounding the sodium ions in ACF films. The ^23^Na NMR revealed that water molecules remained in the Na-ACF films after drying. Linking the bound water around Na^+^ ions to manipulate their electrical properties is a challenging endeavour.

## Results and discussion

### Effect of temperature on storage capacity

**Figure 1:**
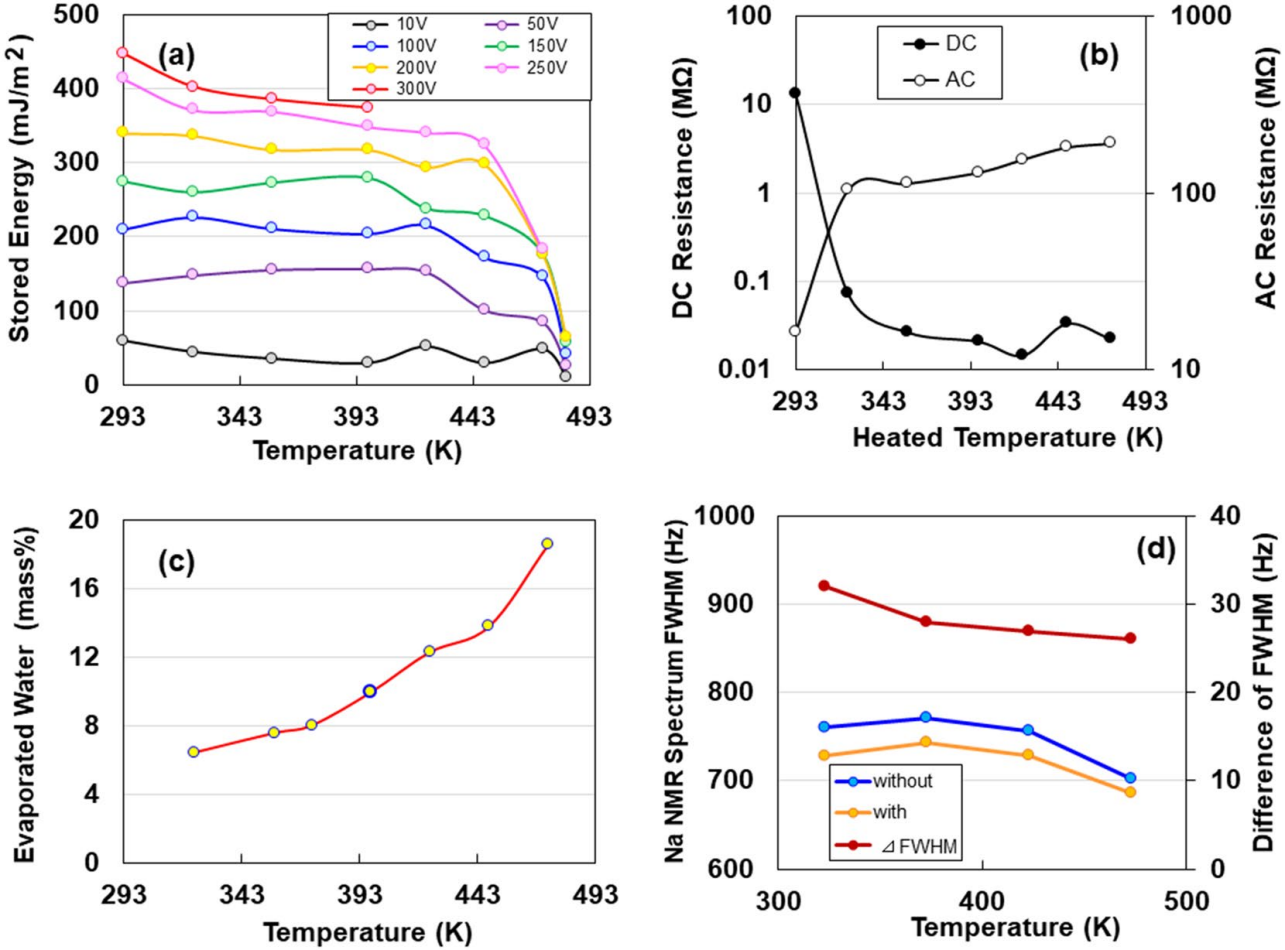
Electric behaviours for dry cellulose nanofibers (Na-ACF) devices heated at 323, 357, 398, 423, 448 and 473 K. (**a**) The discharging behaviours for a constant current of 1μA after 2 mA-250 V charging for 5 s. (**b**) The stored energies by heating treatment for 600 s. (**c**) Alternating current (AC) resistance and evaporated water content for heating treatment. (**d**) Temperature dependence of full-widths at half maximum (FWHM) of the peaks in the solid-state ^23^Na NMR spectra with and without ^1^H decoupling, and their differences of full-width at FWHM of the peaks.

Because Na-ACF with Na carboxylate ions exhibited a higher storage effect than metal (M)-ACF with other M radicals^2^, we used Na-ACF for all experiments. From discharging curves (Supplementary Information [hereafter, referred to as (SI)], Fig. S1), after 2 mA-10 V charging at 250 V for 5 s using Na-ACF devices heated at 323, 373, 423, and 473 K for 600 s. The temperature dependency of the storage capacity for these devices is shown in Fig. 1a, as a function of voltage applied at 10, 50, 100, 150, 200, 250, and 300 V. The CNF specimens changed from transparent to very light brown, and lost toughness when heated above 473 K. Heating to 448 K without discoloration was attributed solely to the dissipation of moisture. The storage capacity decreased gradually till 443 K but decreased rapidly above that temperature and reached almost zero at temperatures above 483 K, suggesting water evaporation from the Na-ACF samples with bound waters. Except for the samples at room-temperature, they could not be stored at charge voltages of 300 V or higher. Figure 1b shows the changes in direct current (DC) electrical and AC resistance at 1 MHz as a function of temperature. The former decreases rapidly as the heating temperature increases and remains almost constant from 323 to 473 K, whereas the latter rapidly increases to 473 K with increasing temperature. This indicated that a change in the equivalent electrical circuit occurred as the heating temperature increased. Water dispersal measurements were subsequently performed to observe water evaporation from the CNF samples owing to heating. The results are shown in Fig. 1c. The moisture dispersal increased with increasing temperature up to approximately 373 K (SI Fig. S3). This indicated that water evaporation caused changes in the CNF organisation. Ohashi et al.^11^ studied the structural and dynamic properties of Na^+^ ions and the water molecules surrounding these Na^+^ ions in cellulose nanocrystals (CNC) using solid-state ^23^Na NMR spectroscopy. Subsequently, they determined the chemical structure surrounding ^23^Na and the motions of the Na nucleus. They found that Na^+^ cations, which were well-hydrated in CNC films, were more dynamic in disordered structures than in ordered structures. Notably, Leu et al.^12^. showed ligand exchange from primary carboxyl groups to Rh_2_(OOCCF_3_)_4_ at the C_6_ position on the CNC surface for the TEMPO-mediated oxidation of CNF. Here, by analogy, we inferred that there were linkers of carboxyl groups, COONa (H_2_O)n (n = 1–4), coordinated to Na on the surface of the CNF, accompanied by chemical shifts (SI Fig. S4). As shown by the ^23^Na peak spectra in CNF specimens heated from 323 to 473 K (SI Fig. S5), no clear difference was identified in the line-shape analysis of the spectra at different temperatures. This indicated that no change in the chemical structure of ^23^Na upon heating from 323 to 473 K occurred. Figure 1d shows the temperature dependence of the differences between the full width at half maximum (FWHM) of the peaks in the solid-state ^23^Na NMR spectra with and without ^1^H decoupling. The difference in the FWHM with and without ^1^H decoupling decreased as the temperature increased. This suggested that the heteronuclear dipolar interactions between ^1^H nuclei in the water molecules and ^23^Na ions decreased moderately with the increasing temperature^11^, resulting in an increase in the motion of ^23^Na ions due to the evaporation of water.

### Infrared (IR) and near-infrared (NIR) spectroscopic analyses

**Figure 2:**
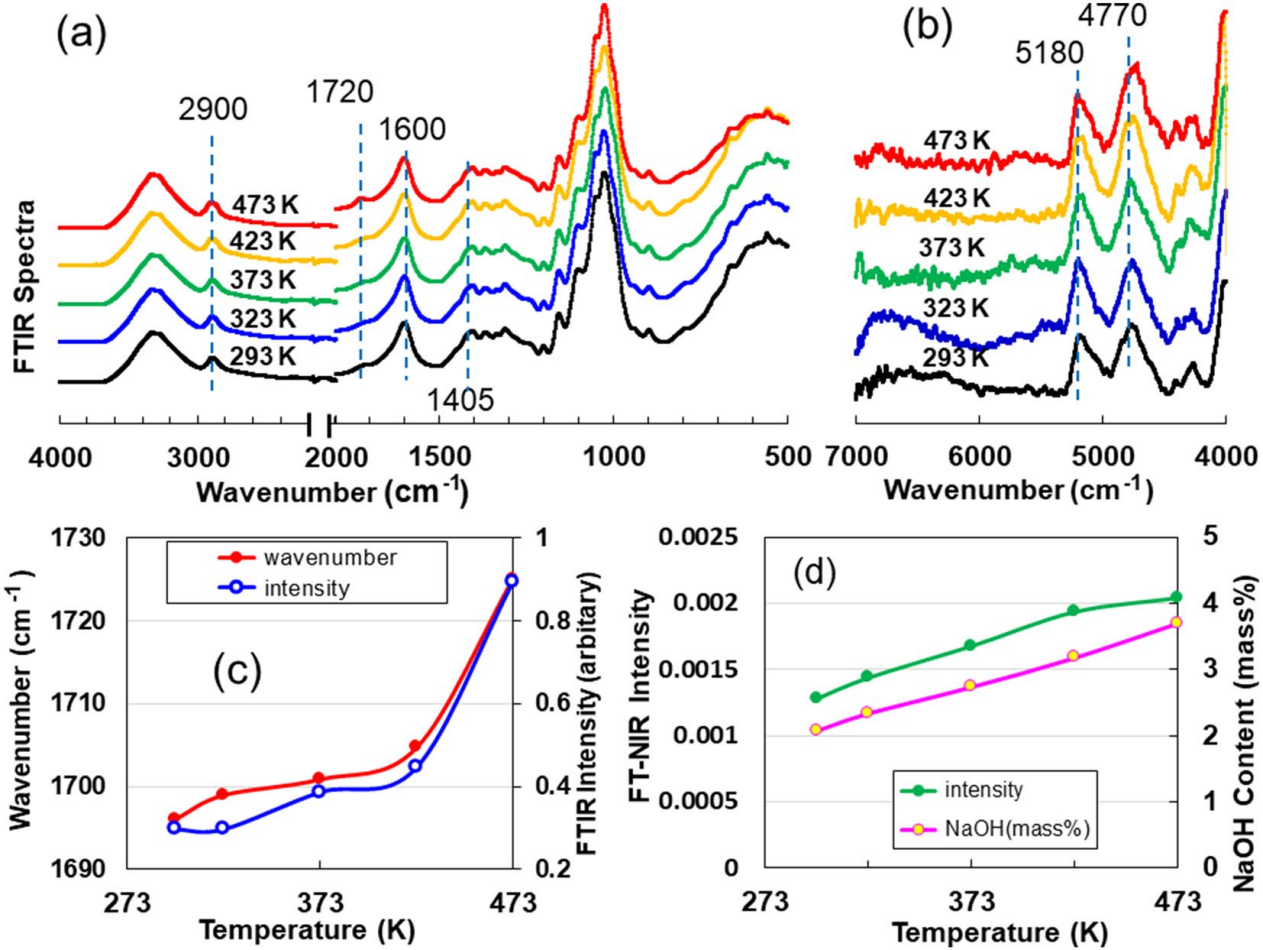
Infrared (IR) and near-infrared (NIR) spectroscopic analyses for dry cellulose nanofibers (Na-ACF) specimens heated at 323, 373, 423 and 473 K. Fourier transform infrared (FT-IR) (**a**) and Fourier transform near-infrared (FT-NIR) (**b**) spectra of Na-ACF films. (**c**) Wavenumber shift and FTIR intensity of 1,720 cm^-1^ after heat treatment. (**d**) FTIR intensity of 4,770 cm^-1^ and content of sodium hydroxide (NaOH) after heat treatment.

IR and NIR spectroscopic analyses were performed to determine the effect of heat treatment on bound water. Figure 2a shows the Fourier Transform Infrared (FTIR) spectra of the heated ACF specimens. The absorption peak intensities at 1600 and 1405 cm^−1^, due to the C = O stretching vibration of carboxylate groups and C–O stretching of the dissociated carboxyl group^13^, respectively, in the ACF did not change with the heating temperature. However, as seen in Fig. 2c, the peak intensity and position of the C = O stretching vibration of the free carboxyl groups at 1720 cm^−1^ increased with increasing heating temperature. Furthermore, there was a characteristic absorption peak at approximately 2,900 cm^−1^ common to all heating temperatures, due to the CH_2_ asymmetric stretching vibration^13^ and complex hydrogen bonds attached to Na^+^ ions^14^. However, the quantitative determination of sodium hydroxide (NaOH) could not be carried out from this absorption peak because of the mingling of the two effects. Conversely, the results of NIR spectra versus heating temperature are shown in Fig. 2b, showing two peaks of approximately 4700 and 5180 cm^−1^. The latter peak corresponded to the coupled tone absorption (stretching + angular change) of the hydroxyl (OH) group. Nishimura^15^ reported that low-frequency absorption appears at approximately 4700 cm^−1^ as the NaOH ratio increases. The 4700 cm^−1^ peak indicated the presence of water represented by complex hydrogen bonds attached to Na^+^ ions. We calculated the NaOH content from the peak intensities of the heated CNF specimens, using the intensity-NaOH content curve (SI Fig. S6) in aqueous NaOH solutions containing various amounts of NaOH^15^. The peak intensity and NaOH content versus heating temperature are shown in Fig. 2d. The intensity and NaOH content increased from 0.00128 to 0.00204, and from 2.08 to 3.70 mass% with the increasing heating temperature, respectively. Therefore, the IR and NIR results indicate the presence of water molecules bound to sodium ions in the Na-ACFs.

### Complex evaluation of energy storage by heating

**Figure 3:**
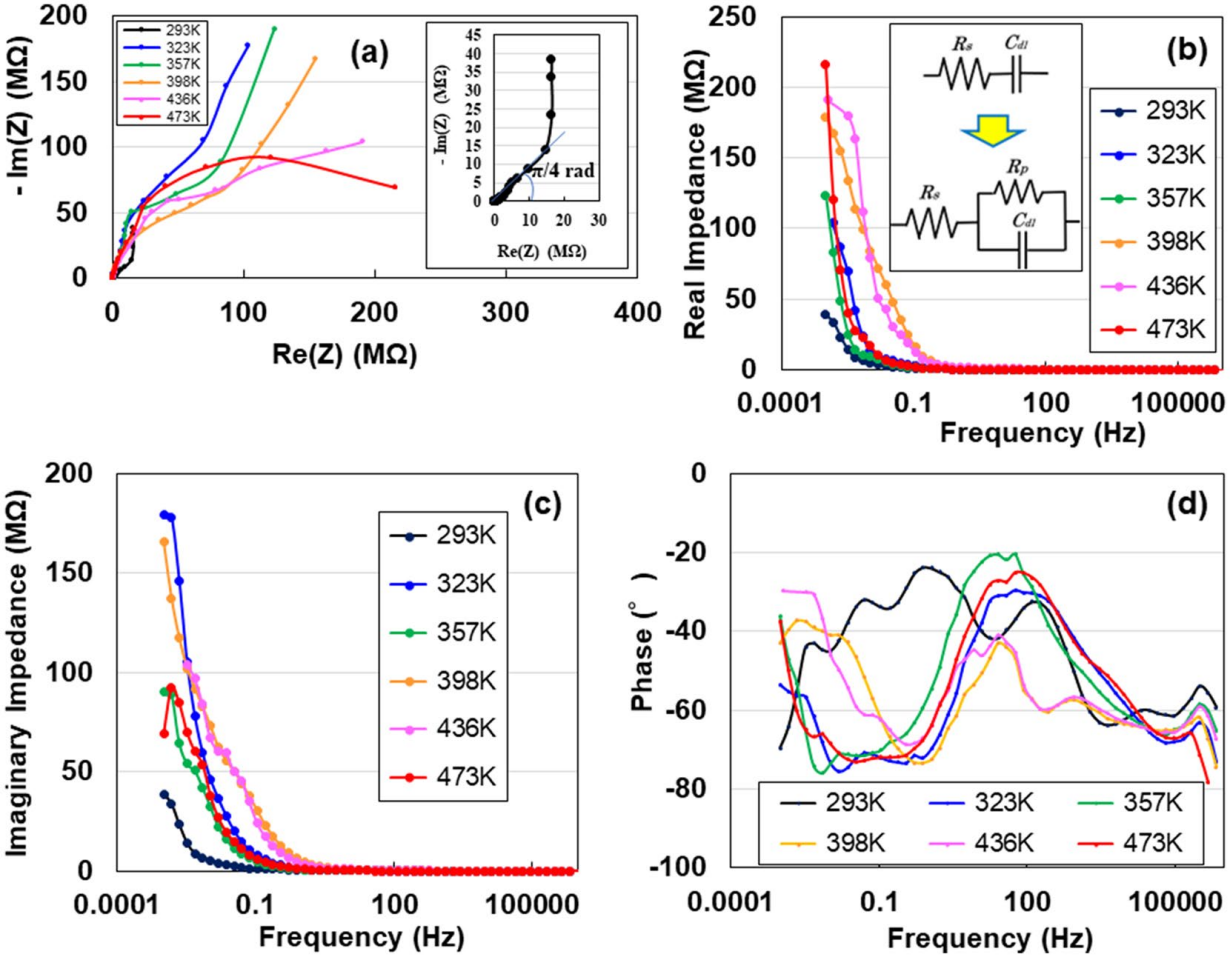
AC impedance analyses. (**a**) Nyquist plot for dry cellulose nanofibers (Na-ACF) devices heated at 323, 357, 398, 436, and 473 K. Real (**b**) and imaginary (**c**) impedances, and phase as a function of frequency.

To investigate the dehydration of water in the heated specimens, we measured the AC impedance from 1 to 1 MHz in the Nyquist and Bode diagrams at 293 K using Na-ACF specimens heated at 293, 323, 357, 398, 436, and 473 K. A Nyquist (complex impedance plane) plot for the specimen as shown in Fig. 3a. The specimen’s variation in impedance with frequency followed two types of the combined patterns: a line with a slope of π/4 rad and a straight vertical line at 293 K (inset of Fig. 3a) and a sharply rising straight line and subsequent parabolic lines at 293, 323, 357, 398, 436, and 473 K. The π/4 rad (Warburg region) is a consequence of the distributed resistance/capacitance in the porous electrode^1,2,16^. As the heating temperature increased, the straight line began to change into a curve, and the Z' component changed more substantially than the Z" component, forming a distorted semicircle. This indicated that the electric double-layer component that contributed to energy storage decreased upon heating. This was proven by the enhancement of the real impedance in Fig. 3b,c of the Bode diagrams and the negative decrease in the phase degree (Fig. 3d) in the lower-frequency region. Figure 3d shows the change in the phase with increasing heating temperature. The reduction to −90°in the phase angle with decreasing frequency in the 293 K- sample was the evidence of DC charging. However, an increase in the temperature changed the structure from simple series to simple parallel circuits, as observed in the increase in the phase in the low-frequency region. The increase in temperature could be caused by the evaporation of water or formation of compounds, particularly when heating above 423 K. Therefore, heating above 423 K removed water from the sample, resulting in the loss of its storage capacity. The changes in the electrical circuitry associated with the sample heating are shown in the insets of Figs. 3b and 1b.

### Optimised structure of ACF with Na-cross-linked hydrogels

**Figure 4:**
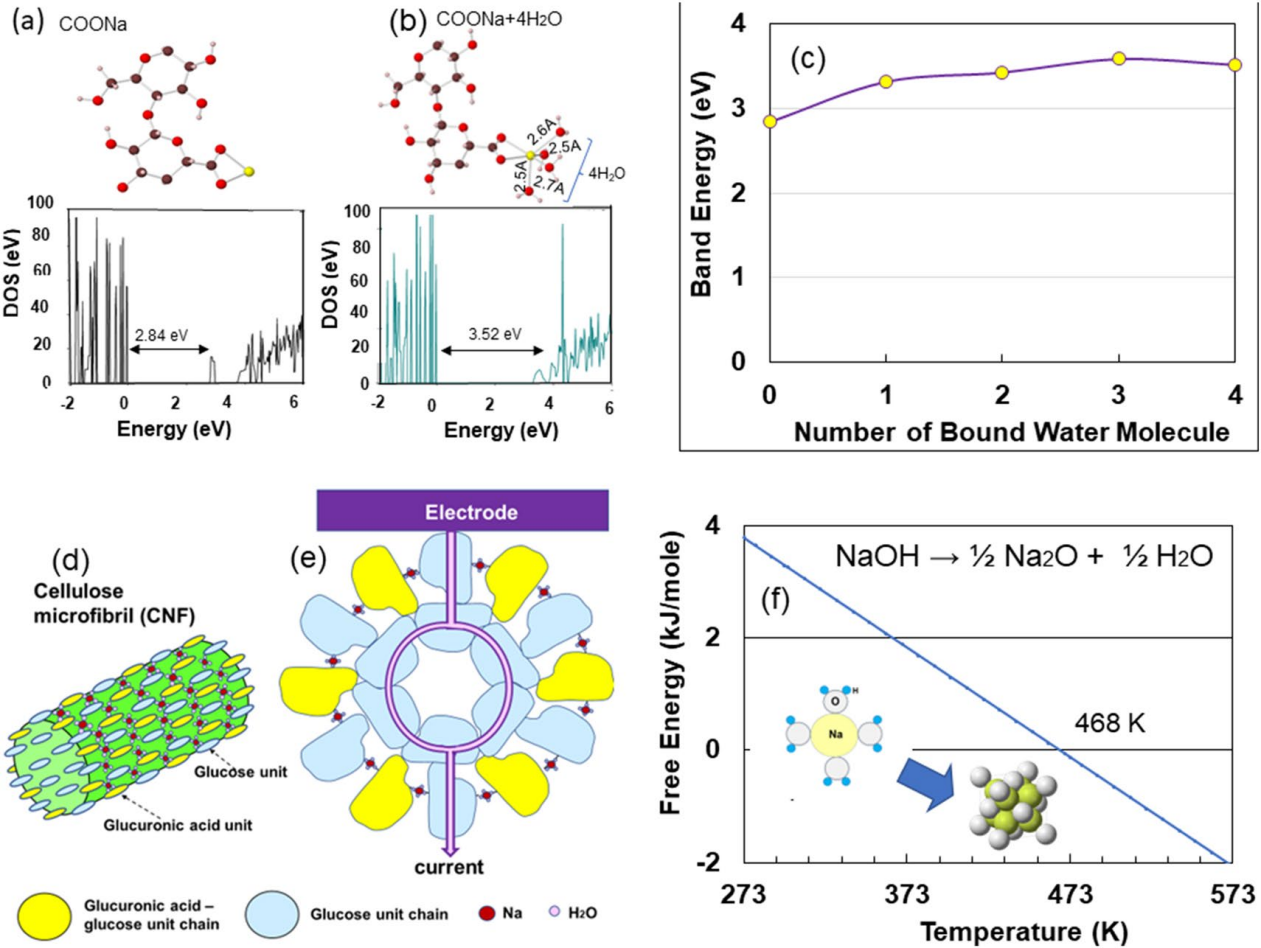
Density of state (DOS) and local structure in COONa-cellulose sheet (**a**) and COONa + 4H_2_O one (**b**). (**c**) Band gap energy for number of bound water molecule. (**d**) Schematic presentation of cellulose mircrofibril with the COONa + 4H_2_O crosslinked between cellulose units and glucuronic ones. (**e**) Cross-section of the microfibril with crosslinking COONa-4H_2_O between the adjacent chains on the surface. (**f**) The relationship between the standard free energy of oxide formation and temperature for Eq. (1). Inset in (**d**): Structure of Na (H_2_O)_4_ cluster and Na_2_O crystal.

In actual TEMPO- oxidized microfibrils crosslinked by electrostatic interactions between Na^+^ ions and negatively charged cellulose fibers^17^, as seen in Figs. 1d and S3, Na^+^ ions in the C_6_ carboxyl groups could have bounded to water molecules on the surface of the microfibrils. To examine the effect of water molecules on the electrical storage, we optimised the local structures around COONa + (H_2_O)n (n = 0–4) using first-principles density functional calculations. We then simulated the density of states (DOS) for the TOCN-COONa (H_2_O)n units, because Nishimura^15,18^ estimated the existence of Na(H_2_O)_4_ in water with a high concentration of Na. The DOSs in the C_12_H_17_O_11_Na- (H_2_O)n celluloses are depicted in Figs. 4a,b and S7. The band gap energy of the localised state of Na^+^ increased with increasing mole number of water molecules and saturates at three molecules of water, as shown in Fig. 4c. The band-gap energy increased owing to the increase in bound water prevents electric leakage from the supercapacitor. This was one of the reasons underlying the remarkable energy storage properties of TOOCN-Na. Therefore, Na-ACF supercapacitors require an Na-metal hydrogel stabilised with water. This is a major difference from electric double-layer capacitors and lithium-ion batteries, which are sensitive to humidity and moisture. Thus, the reason why the stored energy decreases when it reaches 443 K is due to the loss of bound water, leading to electrical leakage from surfaces of cellulose microfibrils. Judging from the discharging behaviours in Fig. 1a and evaporated water content for heating treatment in Fig. 1c, the amount of bound water that is sufficient to yield the desired capacitance for the supercapacitor would be around 14 mass%.

A schematic of a microfibril CNF consisting of glucose and glucuronic acid units with a surface layer crosslinked by Na-carboxy groups with four binding water molecules is shown in Fig. 4d. Figure 4e showed a cross-sectional view of 18 cellulose molecular chains (see SI, S8) whose surface layer consisted of alternating glucose and glucuronic acid units cross-linked by Na carboxy groups with four bound water molecules. In this supercapacitor system, the current during discharge after being released from the electrical circuit, flowed from the positive electrode through the glucose unit molecules of the micro fibrils to the negative electrode.

Monovalent Na^+^ ions readily bounded to water molecules^19^. The hydration complexes, Na (H_2_O)_n_, were stabilised by Na-water interaction^20^. Water molecules in close proximity to Na^+^ ions remained in the Na-CNC films after drying^11^. As the temperature increased, the water molecules gradually evaporated and formed Na_2_O, thereby increasing electrical resistance and drawing the phase angle to zero, consequently degrading the energy storage properties, as shown in Figs. 1b, 3d, and 1a, respectively.

As Fig. 1a showed that the disappearance of storage capacity at temperatures above 423 K was due to water dissociation from hydrogels; thus, we finally considered the interaction between Na^+^ ions and water present in the adjacent cellulose molecular chains on the same fibril surface. Assuming that the presence of Na between cellulose molecules as NaOH, the following reaction occurred upon heating (SI, S9):

1 $${\text{NaOH}} \to {1}/{\text{2Na}}_{{2}} {\text{O}} + {1}/{\text{2H}}_{{2}} {\text{O}}\quad \Delta G = { 9}.{122} - 0.0{195}T$$

The relationship between the standard free energy of oxide formation and the temperature is illustrated in Fig. 4f. Na_2_O and water were formed at temperatures above 468 K, which was consistent with the experimental results shown in Fig. 1a.

## Conclusions

The decrease in the storage capacity of the Na-ACF supercapacitors with increasing heating temperature from 423 K could be explained by the increase in AC resistance and the draw to 0 in the phase angle, owing to the dissociation of bound water in the hydrogels and formation of Na_2_O oxides. Bound water was formed by cross-linking adjacent cellulose molecules as Na(H_2_O)_4_ clusters. Therefore, bound water is necessary for the development of biomaterial electronics. As a result, discharging currents flow from the positive electrode through the glucose unit molecules of the micro fibrils to the negative electrode. Practical use with higher output currents could be obtained by integrating CNF films using a nano-electro mechanical system (NEMS).

## Methods

The TEMPO-mediated oxidation and subsequent mechanical distribution were performed according to a previously reported method^1,2^.

The AC capacitance and DC charging/discharging behaviours were analysed using galvanostatic charge/discharge with a potentiostat/galvanostat (SP-150, BioLogic Science Instruments, France) with a DC voltage of 10 V and a charging current of 1 μA for ~ 60 s and 2 mA for 50 s at 293 K, respectively. The energy stored under the application of voltages ranging from 10 to 500 V was determined using a DC voltage current source/monitor (G247G, ADCMT). The optimised local atomic configurations of the C_12_H_17_O_11_Na and C_12_H_17_O_11_Na-(H_2_O)n units were determined using plane-wave-based first-principles density functional calculations (VASP 5.3)^20^.

### Supplementary Information

Supplementary Information.

## Data Availability

Correspondence and requests for materials should be addressed to M.F. (mikio.fukuhara.b2@tohoku.ac.jp).
