# Supplementary material for: High-energy storage capacity of cellulose nanofiber supercapacitors using bound water
Source: Sci Rep. 2023 Oct 3;13:16600. doi: 10.1038/s41598-023-43222-7 (PMC10547691; doi:10.1038/s41598-023-43222-7)
Supplement: Supplementary file 1 — Supplementary Information. [file 41598_2023_43222_MOESM1_ESM.docx]

**High-energy storage capacity of cellulose nanofiber supercapacitors using bound water**

Mikio Fukuhara,^1,a)^ Tomonori Yokotsuka,^1^ Takuya Takashina,^2^ Nobuhisa Fujima,^3^ Masahiro Morita,^4^ Tatsunori Ito,^4^ and Takeshi Nakatani^4^, and Toshiyuki Hashida^1^

^1^ New Industry Creation Hatchery Center, Tohoku University, Sendai 980-8579, Japan,

^2^ Instrumental Analysis Group, Graduate School of Engineering, Tohoku University 980-8579, Japan,

^3^ Faculty of Engineering, Shizuoka University, Hamamatsu 432-8561, Japan,

^4^ Fuji Innovative Materials Research Laboratory, Nippon Paper Industries, Co. Ltd., Fuji417-8520, Japan,

1. Electronic mail: [mikio.fukuhara.b2@tohoku.ac.jp](mailto:mikio.fukuhara.b2@tohoku.ac.jp)

**S1. Methods**

Once-dried bleached hardwood kraft pulp fibers were used as the cellulose source for 2, 2, 6, 6-tetramethylpiperidine-1-oxyl radical (TEMPO)-mediated oxidation at 293 K under aqueous conditions. TEMPO-mediated oxidation and subsequent mechanical distribution were performed according to a previous reported method^22^. The 0.5% (w/v) TOCN/water dispersion was converted to a film with approximately 5 μm thickness. A 5-μm-thick ACF specimen was fabricated on an Al substrate by electrophoresis method at 10 V and ～0.01 A for 120 s. The TOCN films obtained were dried in a ventilated oven at 323 K overnight. A 5-μm-thick ACF specimen was fabricated on an Al substrate by electrophoresis method at 10 V and ～0.01 A for 120 s.

Measurements of moisture splash from TOCN samples heated at 323, 348, 373, 398, 423, 448, and 473 K for 10 minutes were made using a thermal drying moisture meter (AND, MS-70, Tokyo).

Attenuated Total Reflection Transmission FT-IR and FT-NIR spectra were collected at room temperature over the 4000–650 cm^−1^ and 4000-7000 cm^−1^ regions, respectively, with a resolution of 4 cm^−1^, using a JASCO model FT/IR 6300 and IRT7000 spectrometer. For each sample, 100 and 500 scans were used for FT-IR and FT-NIR, respectively. The TOCN layer for transmission FT-NIR was scraped three times for each sample, for approximately 5 μm. The TOCN shavings scraped off from the surface were sandwiched it with a 3 mm square KBr, then compressed to obtain an approximately 500-μm-thick pellet. All IR and NIR spectra showed the absorbance (A = −log(I/Io)) as a function of the incident wavenumbers.

The CNF-Na films were frozen with liquid nitrogen, ground with a mortar and pestle into flakes, then packed into a plastic drinking straw (5 mm in diameter). All NMR experiments were performed on static samples under atmosphere of a room temperature. Solid-state proton-decoupled ^13^C and ^23^Na NMR spectra were recorded on a JNM-ECZ600R NMR spectrometer operating at 600 MHz. The strength of the radio frequency pulse for ^1^H decoupling was 85 kHz in ^23^Na NMR. ^23^Na chemical shifts were referred to the resonance peak of NaCl.

Because the discharging curves are not generally straight line, the amount of energy stored (*E*) can be calculated from the integral value of the *V* (voltage)-*t* (time) curve under a constant current (*I*): *E* = *I*×$\sum_{t=0}^{t=n} VI$(Joule), where *n* is a measurement point when *V* = 0.

**S2.　Discharging curves of heated specimens**

We measured discharging behaviours after 2mA-10V charging at 250 V for 5 s, using Na-

ACF devices heated at 323, 373, 423 and 473 K for 600 s. The electric storage decreases with increasing of heating temperatures, suggesting water evaporation from Na-ACF samples with bound waters.


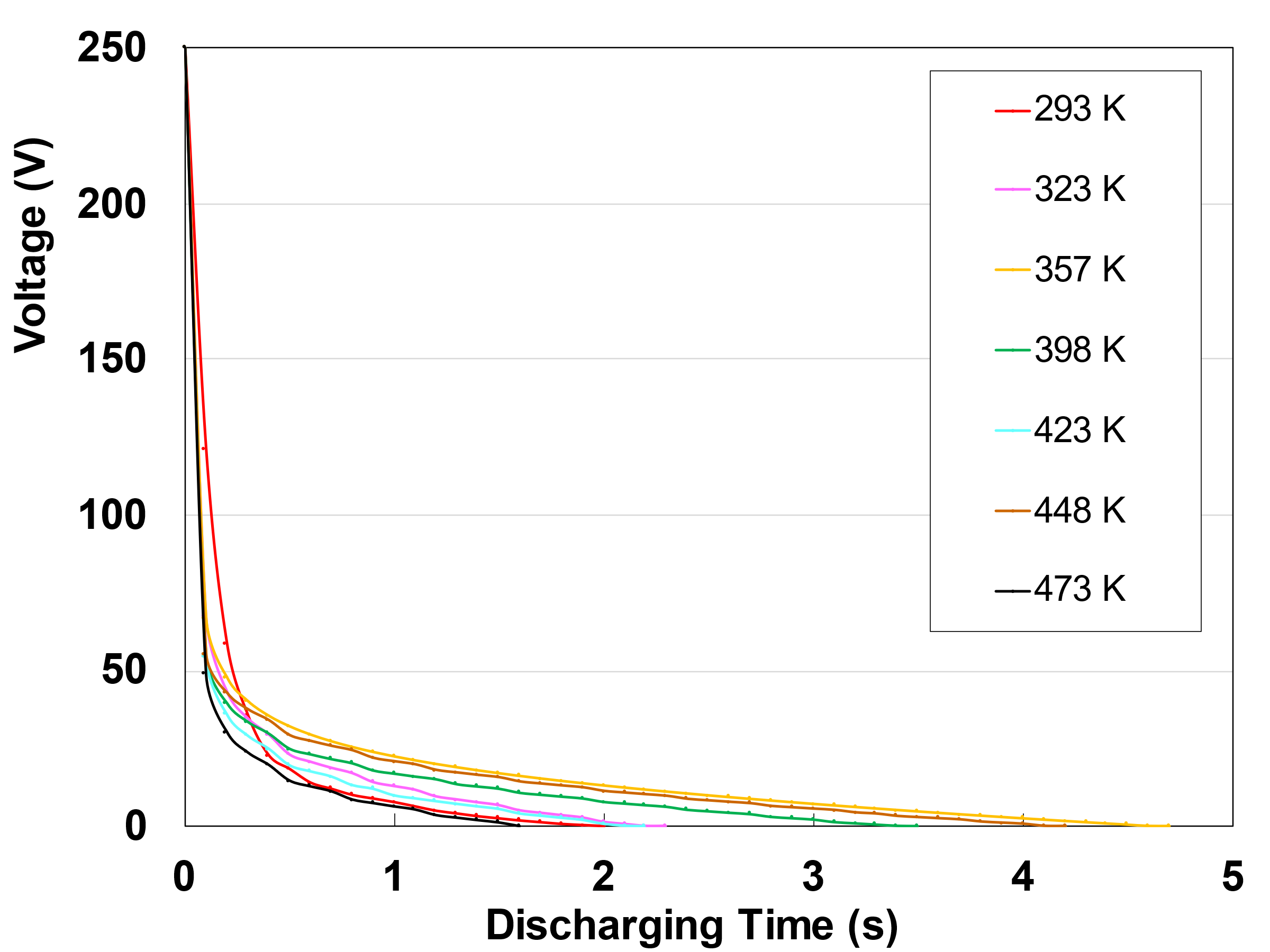


Fig. S1 The discharging behaviours for a constant current of 1μA after 2mA-250V charging for 5 s.

**S3.** **Applied voltage dependency of stored energy on Na-ACFs**

We measured the energy storage properties of Na-CNFs with 1.8 μm. The result is shown in Fig. S2. The stored energy decreases with increasing heated temperatures. The possible voltage for charging also decreases as the temperature rises.

Fig. S2 Applied voltage dependency of stored energy on Na-ACFs.

**S4. Thermal analyses of** **TOCN-COONa specimen**

In order to investigate the thermal degradation behaviours during heating run of the specimen, a differential thermal gravimetric analyzer (TG-DTA) was carried out from 373 to 873 K at a constant heating rate of 0.67 K/s in air, by using of thermogravimetric analyzer (Hitachi STA7200RV). To exclude absorbed water contents, the specimen was first heated from 303 to 373 K at a constant heating rate of 1.67 K/s in nitrogen and then kept for 540 s. In the TGA curve represented by weight (%), Fig. S3 shows weight loss of around 10% up to 373 K due to evaporation of absorbed water. After water evaporation, DTG curve showed prominent thermal degradation from 458 K, along with notable decrease of TGA, leading to deterioration of electric storage.


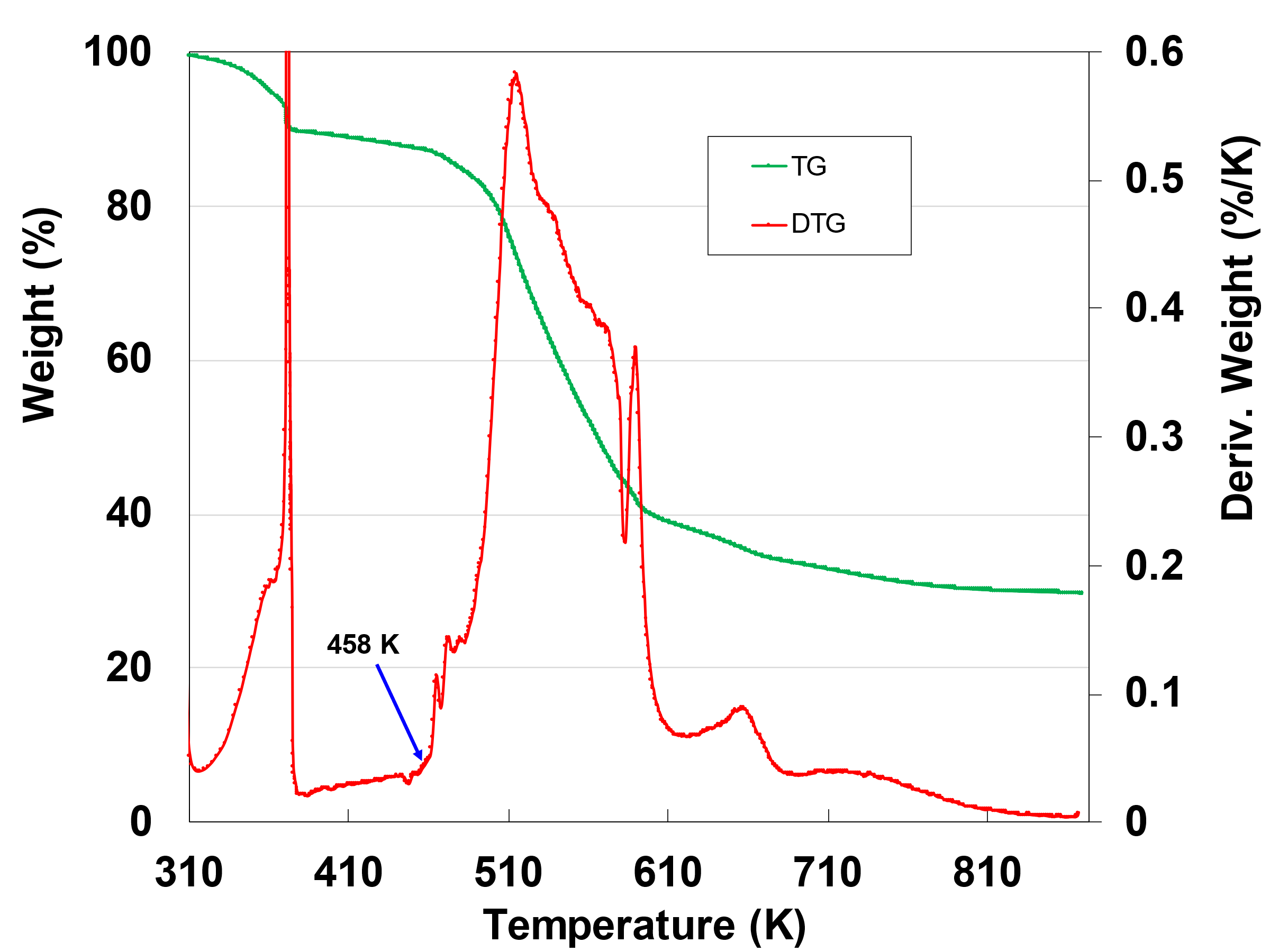


Fig. S3 TGA and DTG curves for Na-ACF specimen.

**S5. ^13^C Solid-state NMR characterization**

In Fig. S4, solid-state ^13^C MAS NMR spectroscopy was employed to analyse the chemical environment of TEMPO oxidized CNF for carboxyl groups. The spectrum of the ^13^C MAS NMR showed broad C_1_, C_4_, C_6_ and C_2_,_3_,_5_ hydroxyl signals^23^ and sodium carboxylate carbon one at about 175 ppm^12^. In addition, a very weak signal with low signal-to- ratio around 184 ppm was observed. Sine similar chemical shifts were observed for the carboxyl carbon atoms of the linkers in the supported Rh_2_(OOCCH_3_)_4_^24^, we assumed a hydrogel (H_2_O)_n_ to sodium on the surface of Na-ACF.


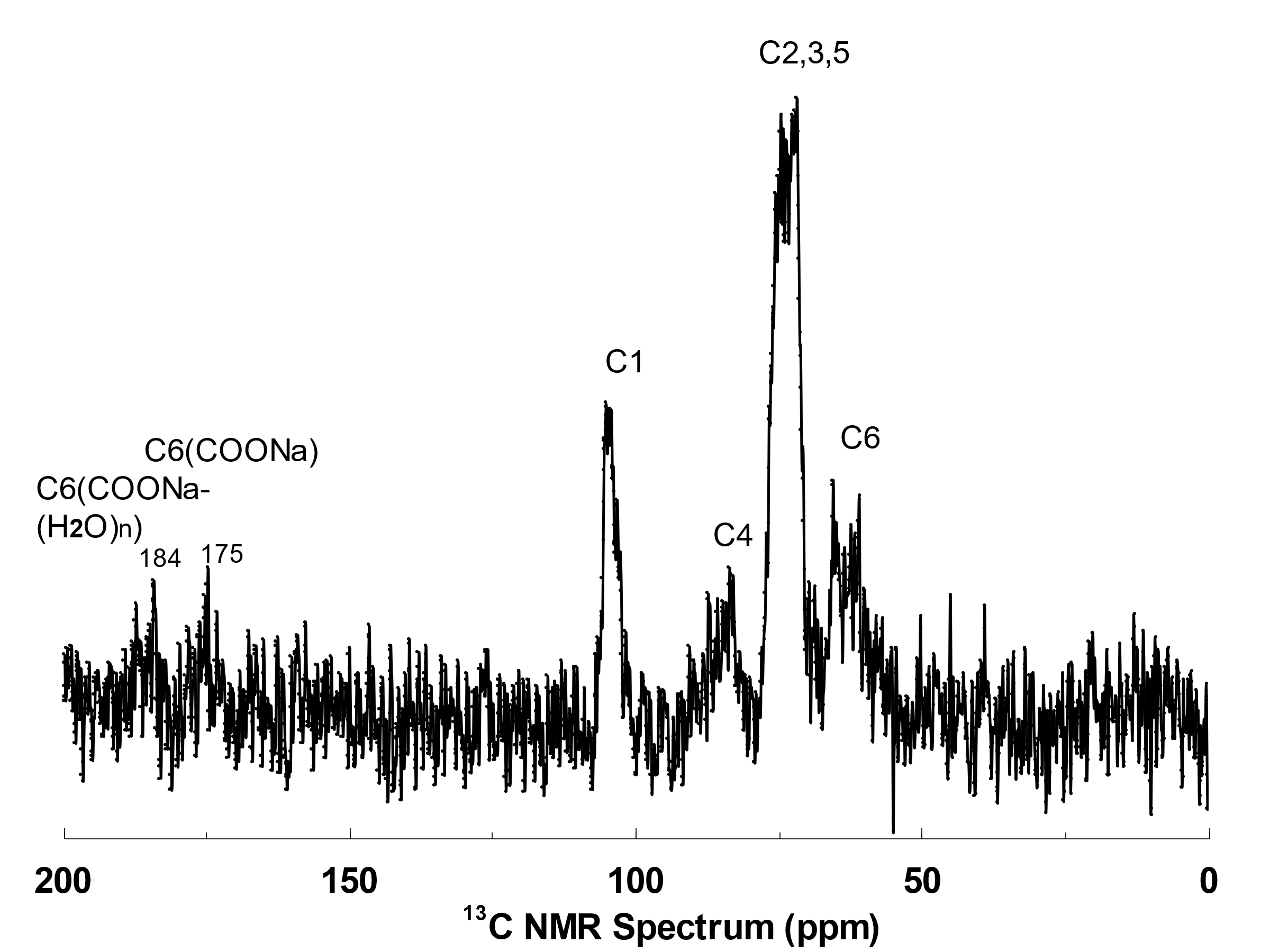


Fig. S4　Solid-state ^13^C MAS NMR spectrum of TOCN-COONa-(H_2_O)n.

**S6. ^23^Na Solid-state NMR characterization**

Solid-state ^23^Na NMR spectra were recorded by a single-pulse technique with and without ^1^H decoupling at 298 K. Both NMR spectra showed single peaks with Lorentzian lineshapes, as shown in Fig. S5. The linewidths of the peaks in spectra obtained without ^1^H decoupling are larger than those obtained with ^1^H decoupling, indicating the presence of heteronuclear dipole interactions between the ^1^H and ^23^Na nuclei. This means ^1^H nuclei are located near the ^23^Na ions.

On the other hand, we could not characterise subpeaks at 0.1 ppm in 373 K- specimens.


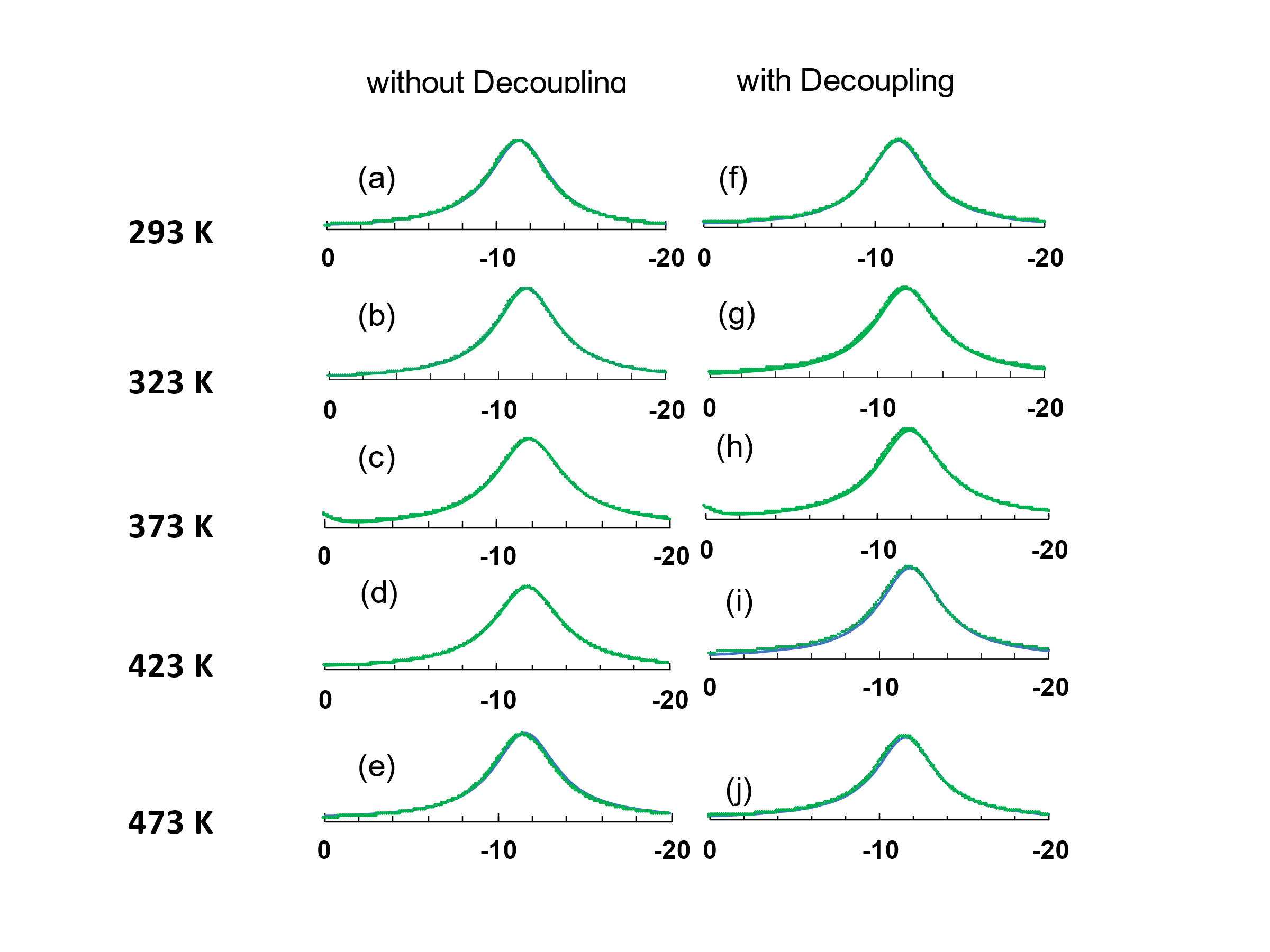


Fig. S5 ^23^Na solid-state NMR spectra of the Na-ACF films. The left five spectra (a), (b), (c), (d) and (e) were recorded without ^1^H decoupling; the right five spectra (f), (g), (h), (i) and (j) were recorded with ^1^H decoupling. The specimens of (b) and (g), (c) and (h), (d) and (i), and (e) and (j) are heated at 323, 373, 423 and 473 K, respectively, using nonheated ones of (a) and (f).

**S7. Calibration curve for NaOH content**

Nishimura^15^ reported a relation between the absorption peak of 4,600 cm^-1^ and NaOH content in high concentration sodium hydroxide solution. The relation is presented at Fig. S6.

Fig. S6 Relation between intensity of 4,600 cm^-1^ and content of NaOH.

**S8. Optimised structure of ACF with Na-cross-linked hydrogels and simulation of the density of states (DOS) for TOCN-COONa-(1, 2, 3) (H_2_O) units**

we optimized the local structures and simulated their DOSs of COONA + (1, 2, 3) (H_2_O) units. Their results are shown in Fig. S7.


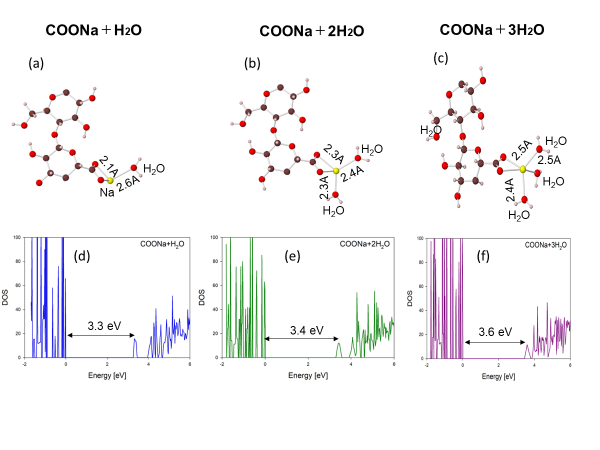


Fig. S7 The structures and their DOSs of COONa+(1, 2, 3)H_2_O: (a) and (d); COONa+H_2_O, (b) and (e); COONa+2H_2_O, (c) and (f); COONa+3H_2_O.

**S9. Cellulose molecular chains comprising cellulose fibrils**

Newman et al.^25^ proposed that single-cellulose fibrils are composed of 18 molecular chains from X-ray and NMR analyses, based on tertiary model of a plant cellulose synthase^26^. Though our TEMPO-oxidized CNFs have an oxidized surface layer, we use the model of Newman et al. to create a cross-sectional view for electrical conduction. A representation figure of a complex assembled from 18 cellulose synthase polypeptides proposed by Newman *et al*. is illustrated in Fig. S8.

+


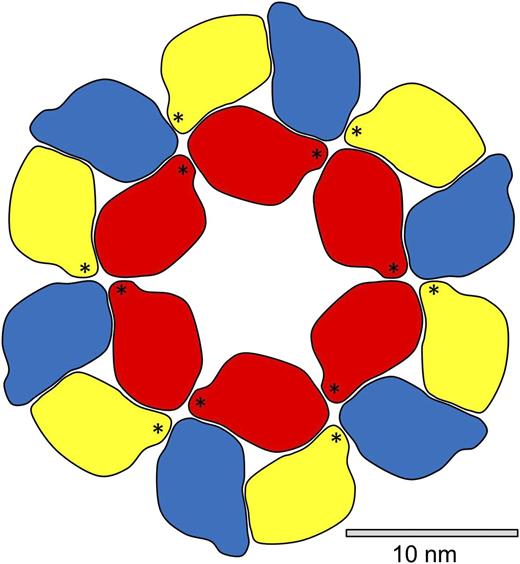


Fig. S8 Model of a cellulose-synthesizing complex assembled from 18 cellulose synthase polypeptides. Outlines are drawn around each cellulose synthase polypeptide in a detailed model of three cellulose synthase particle published by Sethaphong et al.^26^

**S10. The standard free energy for formation of oxide, Na_2_O**

Equation (1) could not be found in the literature, so it was synthesized from Gibbs free energies of each elemental reaction.

We obtain the following reaction from the Na-O-H thermochemical water splitting cycle.^27^

2NaOH + 2Na = 2Na_2_O + H_2_ ∆G = 8.529 - 0.00293 T (S1)

The decomposition reaction of Na_2_O and the synthesis reaction of H_2_ are obtained from the following equations^28^.

Na_2_O = 2Na + 1/2O_2_ ∆G = 23,9540 - 0.008085 T (S2)

H_2_ + 1/2O_2_ = H_2_O ∆G = -14,2400 + 0.00325 T (S3)

From (S1), (S2) and (S3), we get the Eq. (1).

NaOH→1/2Na_2_O+1/2H_2_O ∆*G* = 9.122-0.0195 *T* (1).

**References**

^22^ Shimizu, M., Saito,T. & Isogai, A. Water-resistant and high oxygen-barrier nanocellulose films with interfibrillar cross-linkages formed through multivalent metal ions, *J. Membr. Sci*., **500**, 1-7. http://dx.doi.org/10.1016/j.memsci.2015.11.0002 (2016).

^23^ Isogai, A. Usuda, M. Kato, T, Uryu, T. and Atalla, R. H., Solid-State CP/MAS 13C NMR Study of Cellulose Polymorphs, Macromol., **22**, 3168-3172 (1989).

^24^ Gutmann, T. *et al*., Natural abundance ^15^N NMR by dynamic nuclear polarization: Fast analysis of binding site of a novel amine-carboxyl-linked immobilized dirhodium catalyst, *Chem Eur. J*., **21**, 3798-3805 (2015).

^25^ Newman, R. H., Hill, S.J. and Harris, P. J., Wide-angle X-ray scattering and solid-state nuclear magnetic resonance data combined to test models for cellulose microfibrils in mung bean cell walls, *Plant Physiol*., **163**, 1558-1567 (2013).

^26^ Sethaphong, L., *et al*., Tertiary model of a plant cellulose synthase, *Proc. Natl. Acad. Sci. USA*, **110**, 7512-7517 (2013).

^27^Marques, J. G. O., Costa, A. L. & Pereira, C., Gibbs free energy (∆G) analysis for the Na-O-H (sodium-oxygen-hydrogen) thermochemical water splitting cycle, *Int. J. Hydro. Ener.*, **44**, 14536-14549 (2019).

^28^ Kubaschewski, O, Evans, E.L. I. & Alcock, C. B. *Metallurgical Thermochemistry*, 4^th^ ed., (Pergamon Press, England, 1967).
